# Obeticholic acid and INT-767 modulate collagen deposition in a NASH *in vitro* model

**DOI:** 10.1038/s41598-020-58562-x

**Published:** 2020-02-03

**Authors:** Beatrice Anfuso, Claudio Tiribelli, Luciano Adorini, Natalia Rosso

**Affiliations:** 1 Fondazione Italiana Fegato, AREA Science Park Basovizza, SS14 km 163.5, 34149 Trieste, Italy; 2 Intercept Pharmaceutical, Inc, 10 Hudson Yards 37th Floor, 10001 New York, NY USA

**Keywords:** Hepatology, Experimental models of disease

## Abstract

Pharmacological treatments for non-alcoholic steatohepatitis (NASH) are still unsatisfactory. Fibrosis is the most significant predictor of mortality and many anti-fibrotic agents are under evaluation. Herein, we assessed *in vitro* the effects of the FXR agonist obeticholic acid (OCA) and the dual FXR/TGR5 agonist INT-767 in a well-established co-culture NASH model. Co-cultures of human hepatoma and hepatic stellate (HSCs) cells were exposed to free fatty acids (FFAs) alone or in combination with OCA or INT-767. mRNA expression of HSCs activation markers and FXR engagement were evaluated at 24, 96 and 144 hours. Collagen deposition and metalloproteinase 2 and 9 (MMP2-9) activity were compared to tropifexor and selonsertib. FFAs induced collagen deposition and MMP2-9 activity reduction. Co-treatment with OCA or INT-767 did not affect ACTA2 and COL1A1 expression, but significantly reduced FXR and induced SHP expression, as expected. OCA induced a dose-dependent reduction of collagen and induced MMP2-9 activity. Similarly, INT-767 induced collagen reduction at 96 h and a slight increase in MMP2-9. Tropifexor and Selonsertib were also effective in collagen reduction but showed no modulation of MMP2-9. All tested compounds reduced collagen deposition. OCA exerted a more potent and long-lasting effect, mainly related to modulation of collagen turn-over and MMP2-9 activity.

## Introduction

Obesity prevalence is booming in both hig- and low-income countries has led to a surge in non-alcoholic fatty liver disease (NAFLD), a condition characterized by liver steatosis. It is estimated that around 25% of the population has simple steatosis and due to the increasing number of overweight subjects this figure is dramatically growing. Although simple steatosis is considered a benign reversible condition, around 30% of patients progress to non-alcoholic steatohepatitis (NASH), a more serious condition. NASH is characterized by cellular damage, inflammation and progressive development of liver fibrosis. If the noxious stimuli persist, NASH can progress to more severe liver disease, such as cirrhosis, and hepatocellular carcinoma^1,2^.

Despite increased understanding in the pathogenesis of NAFLD and the identification of numerous therapeutics targets for drug development, no agent is yet approved for this disease.

Progressive fibrosis, cirrhosis and the subsequent liver failure is a consequent of the enhanced deposition of extracellular matrix in the liver, which is the most important cause of liver-related death in patients with NASH^3,4^. Several models describing NASH pathogenesis propose that when the amount of free fatty acids (FFAs) in hepatocytes is overwhelmed, lipotoxic species formation leads to hepatocyte injury, inflammatory cells recruitment, and hepatic stellate cells (HSCs) activation. The massive production of collagen lead to an insufficient degradation of the extracellular matrix^4^, and thus contribute to its deposition.

The Farnesoid X Receptor (FXR), a ligand-activated nuclear transcription factor, represents a promising therapeutic target for NASH^5^. FXR is the primary sensor for endogenous bile acids and plays a crucial role in glucose and lipid homeostasis in the liver^6,7^. FXR is down-regulated in patients with NAFLD and its activation exerts beneficial effects counteracting obesity, steatosis, inflammation, and fibrosis^5^. Due to these favorable effects in chronic liver disease, several small molecules activating FXR are being developed^7,8^.

Obeticholic acid (OCA, INT-747) is a bile acid-derived FXR agonist currently in phase III trials for the treatment of NASH and has already shown its potential for treating hepatic steatosis, inflammation, and fibrosis while increasing insulin sensitivity^9–11^. Currently, two on-going phase III trials are evaluating OCA effects in non-cirrhotic NASH patients with stage 2 and 3 fibrosis (REGENERATE)^12^ and in compensated cirrhotic patients (REVERSE)^13^.

The dual FXR/TGR5 agonist INT-767 has also raised interest in the treatment of NAFLD/NASH. In addition to efficacy against chronic cholangiopathies^14^, INT-767 improves histological features of NAFLD/NASH, reduces hypercholesterolemia, visceral adipose tissue accumulation and the development of atherosclerosis in rodent models^15–20^.

With the increasing interest in the development of anti-fibrotic drugs and the rapid development of new therapeutic agents, innovative *in vitro* tools are needed to screen new potential drugs. We have previously developed an *in vitro* human NASH model able to reproduce the initial phases of NASH development due to cell-to-cell interactions^21^. Specifically, we reported that exposure to FFAs induced their accumulation into hepatocytes and the activation of hepatic stellate cells, as demonstrated by over-expression of alpha-SMA, accumulation of extracellular collagen, and modulation of metalloproteinase (MMP) and tissue inhibitor of metalloproteinase (TIMP)^22^.

In the present study, we employed this model to evaluate the anti-fibrotic properties of OCA and INT-767. In particular, we demonstrate that both compounds are able to reduce collagen deposition through the modulation of collagen degradation. When compared to other molecules, tropifexor and selonsertib, currently being tested in clinical trials for treatment of NASH, we observed that OCA exerted the most potent and long-lasting effect, reducing collagen content by about 50% compared to FFAs-treated controls.

## Materials and Methods

### Chemicals

Cell culture medium Dulbecco’s modified Eagle’s high glucose medium (DMEM-HG), L-glutamine, penicillin/streptomycin, fetal bovine serum and EuroGOLD RNA pure were purchased from Euro-clone (Milan, Italy). 3-(4,5-dimethylthiazol-2-yl)-2,5-diphenyltetrazolium bromide (MTT), dimethyl sulfoxide (DMSO), oleic acid (C18:1), palmitic acid (C16:0), phosphate-buffered saline (PBS) were obtained from Sigma Chemical (St. Louis, MO, USA). Reagents for cDNA synthesis and iQ SYBR Green Supermix were from Bio-Rad Laboratories (Hercules, CA, USA). AlphaLISA cell lysis buffer and Alpha Immunoassay buffer were from Perkin Elmer (Boston, MA, USA). Obeticholic acid (INT-747, OCA), INT-767, tropifexor, and selonsertib were kindly provided by Intercept Pharmaceuticals, Inc. (New York, NY, USA).

### Cellular *in vitro* model and treatment

Hepatic stellate cells (HSCs) LX2 were kindly provided by Dr. Scott Friedman (Mount Sinai School of Medicine, New York, NY). The hepatic cell line Huh7 (JHSRRB, Cat#JCRB0403) was obtained from the Japanese Health Science Research Resource Bank (Osaka, Japan). Both cell lines were maintained in DMEM-HG supplemented with 1% v/v fetal bovine serum, 100 U/mL penicillin/streptomycin, and 2 mM L-glutamine at 37 °C in 5% CO_2_ air humidified atmosphere.

The simultaneous co-culture (SCC) was freshly prepared in cell ratio 5:1 (hepatocytes:HSCs) for each experiment. For the induction of NASH, SCC and monoculture were exposed to 1200 µM of free fatty acids (FFAs) (oleic:palmitic ratio 2:1) as previously described^22^ and co-treated with OCA, INT-767, tropifexor, or selonsertib at 24, 96, and 144 hours. Medium containing FFAs and drugs was refreshed every 2 days until they reach the experimental time point. Effect of tested drugs was evaluated also in absence of FFAs to assess possible side effects. The maximal concentration of vehicle (DMSO) was 0.22% v/v. In order to guarantee a final 80–90% cell confluency the initial cell seeding density was 20,000 cells/cm^2^, 10,000 cells/cm^2^, and 5,000 cell/cm^2^ at 24, 96 and 144 hours respectively. Even if the initial cell seeding densities were different for each experimental time, the number of cells at the end of each time was the same (Supplementary Fig. S1A,B). Any eventual over-confluence was discarded by microscope observation of each well, as shown in the representative pictures reported in Fig. S1C. Each experimental condition was seeded in triplicate and considered as a single biological replicate. If not differently stated, the data represent the mean ± standard deviation (SD) of at least three independent biological replicates, using different cell batches, and different media preparation with the supplementation with drugs; FFAs; or vehicle.

### Cell viability

The cytotoxic effect of FFAs and compounds alone or in combination was assessed by MTT colorimetric assay at 144 hours. SCC was plated at 5000 cell/cm^2^ and treated with increasing concentration of each compound (0–100 µM). Also Hepatocytes and HSCs were plated at the same density and tested with OCA and INT-767 0.1, 1, and 10 µM. After 144 hours, the medium was removed and cells were incubated for 1 hour with MTT at the concentration of 0.5 mg/ml. Afterward, the medium was removed and formazan crystals were dissolved in 200 µl of DMSO. 100 µl from each well were moved to a microtiter plate and optical density (OD) was determined at 562 nm wavelength on an Enspire® Multimode Plate Reader (Perkin Elmer, Waltham, MA USA).

### RNA extraction, cDNA synthesis, and gene expression analysis by qPCR

Total RNA was isolated using EuroGOLD RNA Pure (EuroClone S.p.A, Milan, Italy) according to the manufacturer’s instructions. The total RNA concentration was quantified spectrophotometrically at 260 nm in a Beckman Coulter DU®730 spectrophotometer (Fullerton, CA, USA) and purity was evaluated by measuring the ratio A260/A280.

Total RNA (1 μg) was reverse transcribed using the High Capacity cDNA Reverse Transcription Kits (Applied Biosystems, USA) according to the manufacturer’s suggestions in a Thermal Cycler (Gene Amp PCR System 2400, PerkinElmer, Boston, MA, USA). Quantitative PCR was performed in a CFX connect^TM^ system (Bio-Rad, Hercules, CA USA). All primer pairs were designed using the software Beacon Designer 8.12 (PREMIER Biosoft International, Palo Alto, CA, USA) and were synthesized by Sigma Genosys Ltd. (London Road, UK). PCR amplification was performed in 15 μL reaction volume containing 25 ng of cDNA, 1 × iQ SYBR Green Supermix and 250 nM gene specific sense and antisense primers and 100 nM primers for 18S. Primer sequences were as follow: 18S (5′-TAACCCGTTGAACCCCATT-3′; 5′-CCATCCAATCGGTAGTAGCG-3′), HPRT (5′-ACATCTGGAGTCCTATTGACATCG-3′; 5′-CCGCCCAAAGGGAACTGATAG-3′), ACTA2 (5′-TGTGAATGTCCTGTGGAATTATGC-3′; 5′-ACACATAGGTAACGAGTCAGAGC-3′), COL1A1 (5′-CGGAGGAGAGTCAGGAAG-3′; 5′-ACACAAGGAACAGAACAGTC-3′), FXR (5′-GCTATGTTCCTTCGTTCA-3′; 5′-ATTCATCAGAGATACCACTATT-3′), SHP (5′-TCCTCTTCAACCCCGATGTG-3′; 5′-CCAGGGTTCCAGGACTTCAC-3′). 18 S and HPRT were used as reference genes. The data were analyzed using the Bio-Rad CFX Manager (version 3.1).

### Quantification of extracellular collagen deposition

After 96 and 144 hours of treatments, cells were lysed in AlphaLISA lysis buffer (Perkin Elmer, Waltham, MA USA) and stored at −80 °C until use. Cell lysates were diluted 1:10 in Alpha Immunoassay buffer and collagen content was quantified using COL1A1 AlphaLISA Detection kit (Perkin Elmer, Waltham, MA USA) according to the manufacturer’s instructions. Results were obtained by fitting data to a dose-response sigmoidal curve generated with Graph Pad Prism® version 5.01 and normalized to total protein.

### MMP2/9 activity

Metalloproteinase 2 and 9 activity was tested at 96 and 144 hours in the supernatant of treated SCC with InnoZyme^TM^ Gelatinase activity assay kit (Calbiochem-Millipore, Darmstadt, Germany). Briefly, 30 µl of supernatant was added into a 96-well plate with 60 µl of activation buffer and 10 µl of substrate working solution. After 2 hours of incubation at 37 °C, the developed fluorescence was read at 405 nm (excitation wavelength of 320 nm) on an Enspire® Multimode Plate Reader (Perkin Elmer, Waltham, MA USA). Obtained data were fitted to a linear standard curve and normalized to the total protein of the respective cell lysate.

### Total protein quantification

Total proteins were quantified by fluorescamine, a non-fluorescent molecule that reacts readily with primary amines in amino acids and peptides to form stable, highly fluorescent compounds. 10 µl of fluorescamine at 4 mg/mL in DMSO was mixed with 40 µl of cell lysate diluted 1:2. Fluorescence was read at 460 nm (excitation wavelength of 390 nm) in an EnSpire® Multimode Plate Reader (Perkin Elmer, Waltham, MA USA). Total protein was calculated using a BSA standard curve dilution.

### Statistical analysis

Statistical analyses were performed using InStat software Version 3.05 (GraphPad Software, Inc., La Jolla, CA, USA). Data are represented as mean ± SD of at least three independent biological replicates. The student’s t-test was performed for statistical comparison between groups. Value of p < 0.05 was regarded as statistically significant.

GraphPad Prism Version 5.0 (GraphPad Software, Inc., La Jolla, CA, USA) software was used to generate graphs.

## Results

### Determination of experimental compound concentration

Survival curves were constructed to select the experimental concentrations of the compounds. Briefly, SCC was exposed to 1200 µM FFAs in the presence of increasing concentrations (0–100 µM) of the drug for 144 hours to identify concentrations with toxic effects. Likewise, cells were also exposed to CTRL medium with increasing concentrations of compounds only to assess their toxicity in the SCC system without FFAs.

**Figure 1:**
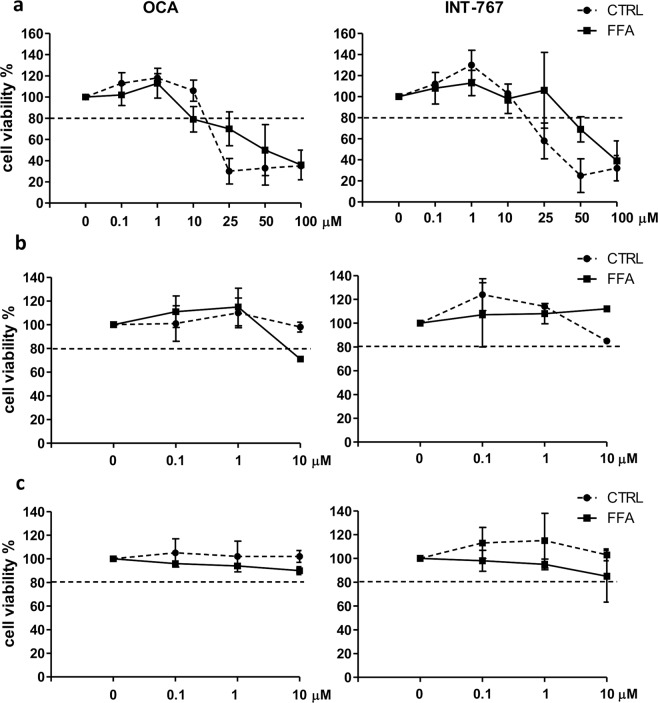
Dose-response curves for OCA and INT-767. (**a**) Simultaneous co-cultures (SCC) were treated with increasing concentrations of OCA (left panel) or INT-767 (right panel) with (full line) or without (dashed line) FFAs for 144 hours. Monocultures of hepatocytes (**b**) and HSCs (**c**) were treated with the selected concentrations of OCA (left panel) or INT-767 (right panel) with (full line) or without (dashed line) FFAs for 144 hours. Horizontal dashed bars indicate the cutoff. Data is represented as mean ± SD of at least three independent biological replicates.

A reduction in cell viability of more than 20% was considered as the exclusion criteria. The cutoff concentration meeting this requirement was 10 µM for both OCA and INT-767. Consequently, the selected experimental concentrations were 10, 1, and 0.1 µM (Fig. 1a).

To evaluate the effect of the selected concentrations on each cell type, the cell viability was assessed also in the monoculture of hepatocytes and HSCs. As shown in Fig. 1b,c, cell viability was higher than 80% for both compounds with the exception of OCA 10 µM, which induced, in the presence of FFAs, a 30% of reduction in hepatocytes viability.

### Effect on stellate cells activation

**Figure 2:**
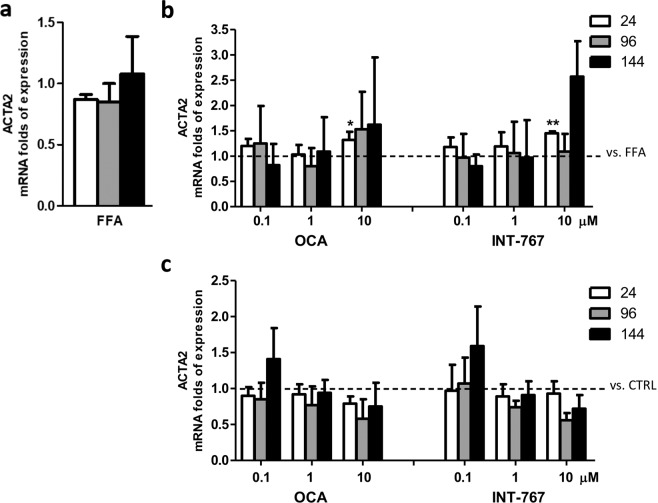
Activation of HSCs. (**a**) ACTA2 mRNA expression upon FFAs exposure vs. CTRL. (**b**) ACTA2 expression following treatment with FFAs and FXR agonists vs. FFAs (dashed bar). (**c**) ACTA2 expression following OCA and INT-767 treatment vs. CTRL (dashed line). Data is represented as mean ± SD of at least three independent biological replicates. *p < 0.05; **p < 0.01 vs. FFAs (**b**) or CTRL (**c**).

We previously reported that exposure to FFAs in SCC induced an up-regulation of ACTA2 (α-SMA), an HSC activation marker, after 24 hours^22^. Unexpectedly, in this study we did not observe such an increase at 24 hours, but rather a trend of up-regulation in mRNA expression of ACTA2 after FFAs exposure compared to vehicle control at longer exposure times (144 hours) (Fig. 2a). Moreover, the addition of drugs did not alter this expression, with the exception of 10 µM for both OCA and INT-767 that showed a significant induction after 24 hours (p < 0.05 and 0.01, respectively), an effect which was not sustained over the time (Fig. 2b). Similarly, the compounds alone did not modulate ACTA2 expression at any time and dose tested (Fig. 2c).

### Effect on collagen deposition

**Figure 3:**
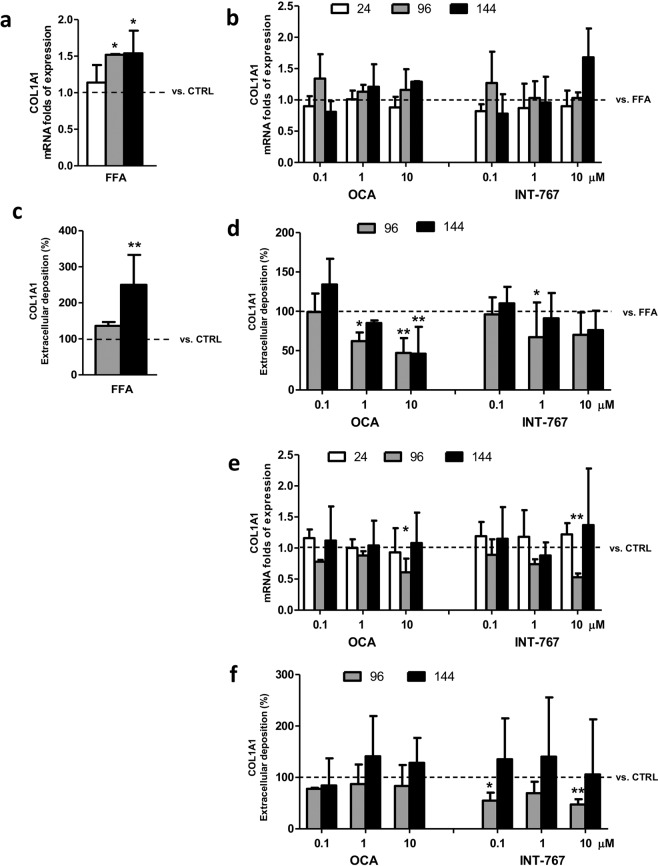
Collagen biosynthesis. (**a**) Col1A1 gene expression upon FFAs exposure vs. CTRL (dashed bar). (**b**) Col1A1 mRNA expression following treatment with FFAs and FXR agonists vs. FFAs (dashed bar). (**c**) Quantification of extracellular COL1A1 deposition upon FFAs exposure vs. CTRL (dashed bar). (**d**) Quantification of extracellular COL1A1 deposition following treatment with FFAs and FXR agonists vs. FFAs (dashed line). (**e**) Col1A1 gene expression upon FXR agonists treatment vs. CTRL (dashed line). (**f**) Quantification extracellular COL1A1 deposition upon FXR agonists treatment vs. CTRL (dashed line). Data is represented as mean ± SD of at least three independent biological replicates. *p < 0.05; **p < 0.01 vs. FFAs (**b**–**d**) or CTRL (A-C-E-F).

Type I collagen is one of the major extracellular matrix components and its production and turnover are associated with fibrogenesis^23^. Therefore, we evaluated collagen biosynthesis after exposure to FFAs. In line with our previous data, when compared to controls the gene expression of COL1A1 was significantly up-regulated starting from 96 hours and it was sustained over the time (1.5 folds; p < 0.05 at 96 and 144 hours). Likewise, collagen in terms of protein expression was also increased starting from 96 to 144 hours, when the difference was statistically significant (250%, p < 0.01) (Fig. 3a,c).

Each compound did not induce any significant change in COL1A1 mRNA expression at any time nor concentration tested as compared to FFA treatment (Fig. 3b). Conversely, both compounds were able to reduce the FFA-induced collagen deposition: OCA induced a clear dose-dependent reduction starting from 96 hours, with a 20% (p < 0.05) reduction at 1 µM and to a 50% of reduction (p < 0.01) at 10 µM, persistent also at 144 hours. Also, INT-767 treatment was able to induce a dose-dependent reduction of collagen, but only at 96 hours with 0.1 µM (p < 0.05) (Fig. 3d).

Effects of compounds on collagen deposition were also evaluated in a non-NASH environment. Both OCA and INT-767 10 µM induced a down-regulation of COL1A1 expression at 96 hours (p < 0.05 and 0.01 for OCA and INT-767 respectively) but this effect was abolished at 144 hours (Fig. 3e). The observed down-regulation of COL1A1 gene expression was consistent with a decrease in the extracellular collagen content only for INT-767 (p < 0.01); a significant decrease of collagen was also observed at 0.1 µM (p < 0.01). Also, in this case, the reduction was restored at 144 hours (Fig. 3f).

### Effects on metalloproteinase activity

**Figure 4:**
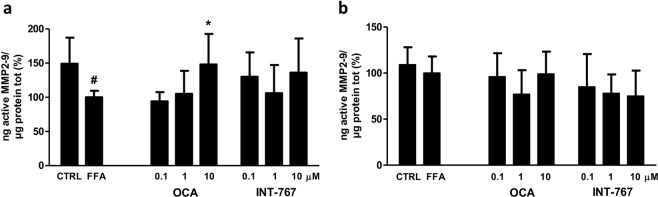
MMP2-9 activity. Enzymatic activity of active MMP2-9 was analyzed in the supernatant of SCC treated with FFAs and FXR agonist compounds after (**a**) 96 and (**b**) 144 hours of treatment. Activity was normalized to the total proteins in the cell lysate and reported as percentage vs. FFAs treated sample. Data is represented as mean ± SD of at least three independent biological replicates. *p < 0.05 vs. FFAs; ^#^p < 0.05 vs. CTRL.

Metalloproteinase 2 and 9 (MMP2-9) are enzymes involved in the extracellular matrix modulation. MMPs are released into the extracellular environment where they contribute to collagen turnover. Their activity is finely regulated both at mRNA and at post-transcriptional level by the interaction with the tissue inhibitor of metalloproteinase proteins (TIMPs)^24^. After 96 hours, MMPs activity was significantly reduced in the cells treated with FFAs (p < 0.05 compared to CTRL). Co-treatment with OCA induced a progressive increase in MMP2-9 activity reaching the control values at 10 µM (p < 0.05 compared to FFAs). At the same time point, INT-767 treatment showed a similar though not significant trend (Fig. 4a).

After 144 hours, despite the clear modulation in collagen deposition (Fig. 3d), MMP2-9 activity was not regulated either by FFAs alone or in combination with the compounds, suggesting that the observed effects on collagen deposition result from earlier events in MMP2-9 modulation (Fig. 4b).

### FXR pathway analysis

**Figure 5:**
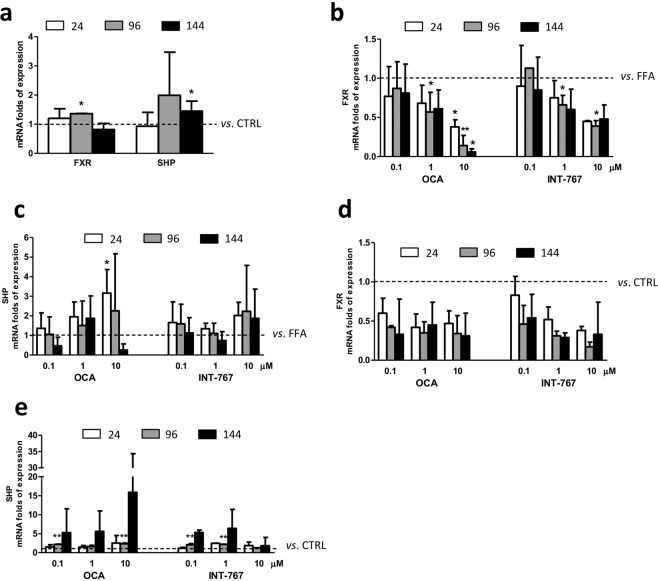
FXR pathway gene analysis. (**a**) FXR and SHP gene expression upon FFAs exposure vs. CTRL. (**b**) FXR gene expression upon FFAs and FXR agonists treatment vs. FFAs (dashed bar). (**c**) SHP gene expression upon FFAs and FXR agonists treatment vs. FFAs (dashed line). (**d**) FXR gene expression upon OCA and INT-767 treatment vs. CTRL (dashed line) (**e**) SHP gene expression upon OCA and INT-767 treatment vs. CTRL (dashed line). Data is represented as mean ± SD of at least three independent biological replicates. *p < 0.05; **p < 0.01 vs. FFAs (**b**,**c**) or CTRL (A-C-D).

FXR agonists activate hepatic FXR to regulate genes involved in glucose and triglyceride metabolism. FXR activation represses gluconeogenesis, TG synthesis, and VLDL export via SHP, a primary FXR responsive gene which contributes to regulate FXR target genes^6^. In this study, we assessed FXR and SHP mRNA expression after exposure to FFAs. As shown in Fig. 5a, FFA exposure induced an increase in FXR expression at 96 hours (p < 0.05 relative to CTRL), whereas at 24 and 144 hours it was almost unchanged. Conversely, the downstream gene SHP was up-regulated after 144 hours (p < 0.05 relative to CTRL). The scenario was more complex when we evaluated the expression of these two genes after treatment with the drugs. While OCA 0.1 µM treatment did not alter FXR and SHP mRNA expression, the higher concentrations induced a dose and time-dependent reduction of FXR expression, especially at 10 µM (p < 0.05 at 24 and 144 hours and p < 0.01 at 96 hours compared to CTRL). Conversely, the expression of SHP was initially up-regulated (p < 0.05 at 24 hours) and then progressively reduced, probably as a consequence of FXR mRNA expression reduction. INT-767 1 and 10 µM treatment was also able to induce a progressive decrease in FXR mRNA expression that was statistically relevant at 96 hours (p < 0.05 and 0.01 at 1 and 10 µM respectively); this modulation was not reflected on SHP expression (Fig. 5b,c).

When cells were exposed only to the compounds, the modulation of FXR and SHP genes was similar to that observed in the co-treatment with FFAs. Although not significantly, FXR mRNA expression was reduced by both OCA and INT-767, while SHP was up-regulated, especially at 96 hours (p < 0.01 with 0.1 and 10 µM OCA and 0.1 and 1 µM INT-767) (Fig. 5d,e).

### Evaluation of collagen deposition and MMP2-9 activity modulation by tropifexor and selonsertib

Recently, several small molecules have been developed and proposed as a treatment for NASH. An easy and predictive model to compare the potential of these compounds in reducing liver fibrosis would be crucial to facilitate screening and to accelerate the selection of the most promising candidates. Thus, we tested our SCC system with other two molecules under evaluation in ongoing clinical trials: a nonsteroidal FXR agonist (tropifexor), and an ASK1 inhibitor (selonsertib).

**Figure 6:**
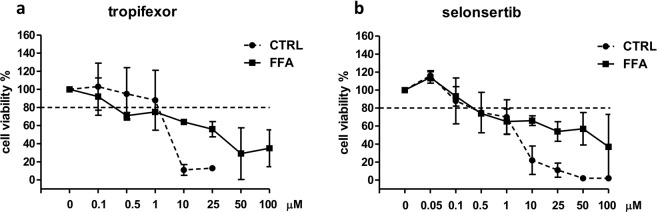
Dose-response curves for tropifexor and selonsertib. Simultaneous co-cultures were treated with increasing concentrations of tropifexor (**a**) or selonsertib (**b**) with (full line) or without (dashed line) FFAs for 144 hours. Horizontal dashed bars indicate the cutoff. Data is represented as mean ± SD of at least three independent biological replicates.

Experimental concentrations were selected based on the same parameters used for OCA and INT-767. The cutoff concentration was 1 µM for tropifexor and 0.5 µM for selonsertib, thus the selected concentrations were 1, 0.5, and 0.1 µM for tropifexor and 0.5, 0.1, and 0.05 µM for selonsertib, respectively (Fig. 6a,b)

**Figure 7:**
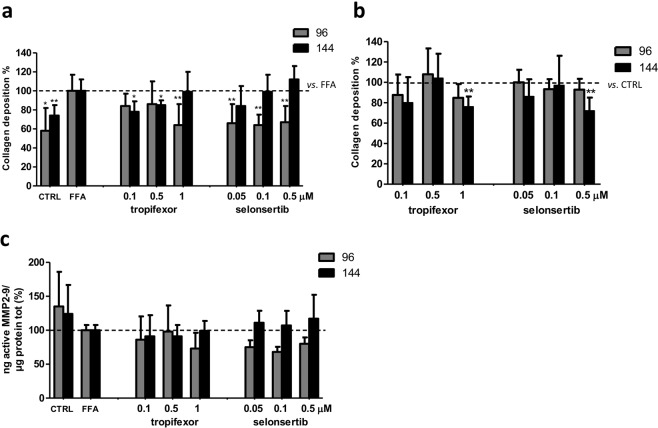
Collagen turnover upon tropifexor or selonsertib treatment. (**a**) Extracellular quantification of the total collagen upon FFAs and FXR agonists treatment vs. FFAs (dashed line). (**b**) or upon FXR agonists treatment vs. CTRL (dashed line). (**c**) Active MMP2-9 was quantified in the supernatant of SCC treated with FFAs and FXR agonists after 96 and 144 hours. Activity was normalized to the total proteins in the cell lysate and reported as percentage vs. FFAs treated sample. Data is represented as mean ± SD of at least three independent biological replicates. *p < 0.05; **p < 0.01 vs. FFAs (**a**) or CTRL (**b**).

Tropifexor treatment was more effective in reducing collagen deposition at lower concentrations (p < 0.01 at 0.1 and 0.5 µM) and at 144 hours, while 1 µM was particularly effective at 96 hours (p < 0.01). At all tested concentrations, selonsertib reduced collagen deposition after 96 hours to the same extent (p < 0.01) but this effect was not sustained since after 144 hours the collagen content was similar to the FFAs samples (Fig. 7a). Treatment with compounds only did not alter collagen deposition at 96 hours, while prolonged exposure induced a significant collagen deposition reduction only at the highest concentrations tested (p < 0.01 with 1 µM tropifexor and 0.5 µM selonsertib) (Fig. 7b).

Regarding MMP2-9 activity, these two compounds did not induce any significant modulation of MMP2-9 at both time points analyzed (Fig. 7c).

## Discussion

The farnesoid X receptor (FXR) is a nuclear receptor that regulates glucose and lipid homeostasis, inhibits inflammation and fibrosis, and thus is a promising target in NASH. In spite of the development of numerous animal models (more than 40 according to the literature)^25,26^, a major limitation to understanding NASH pathophysiology and to assess treatment options is the lack of an adequate preclinical model that fully resembles the human condition. In the current study, we assessed the anti-fibrotic effect of OCA (the most advanced FXR agonist) and INT-767 (a promising dual FXR/TGR5 agonist) in a human co-culture *in vitro* model that reproduces the early stages of fibrogenesis in response to hepatic FFA overload^22^.

The most relevant clinical endpoint in NASH is the development of fibrosis^3,4^ by the activation of HSCs from a quiescent state to a highly proliferative phenotype. This process is characterized by modulation of the cytoskeletal filament, and primarily by enhanced expression of contractile α-SMA filaments together with the production of large amounts of collagen type I, the most abundant ECM protein in cirrhotic livers^27,28^. In our model, we found a significant increase in collagen production at both mRNA and protein levels after exposure to FFAs. Despite unchanged α-SMA gene expression, the significant increase in extracellular collagen content (2.5 times higher than control) indicated activation of HSCs upon noxious stimuli.

OCA was reported to decrease hepatic fibrosis in NASH patients^9,11^. In a multicenter, randomized clinical trial (FLINT study), patients with non-cirrhotic, non-alcoholic steatohepatitis were treated with OCA (25 mg daily) or placebo for 72 weeks. The treatment was associated with a significant reduction of liver fibrosis (35% in OCA-treated vs. 19% in the placebo group, respectively)^9^. Similarly, in a study with patients with type 2 diabetes and NAFLD, markers of liver fibrosis decreased significantly in the group treated with 25 mg OCA^10^. Recently, Feaver *et al*. demonstrated that treatment with OCA 0.5 µM was able to significantly reduce pro-COL1A1 secretion in a complex *in vitro* model composed of primary hepatocytes, stellate cells and macrophages^29^. In line with this observation, in our lipotoxic SCC model, OCA was able to induce a dose and time-dependent reduction in collagen deposition at both 1 and 10 µM, confirming the direct effect of this drug on fibrosis. In the absence of FFAs, OCA did not exert any significant effect on collagen production. INT-767 was reported to reduce collagen deposition and ameliorate fibrosis stage in different diet-induced NAFLD/NASH animal models^15,18,20^. In a comparative study, both OCA and INT-767 significantly improved all histological parameters relative to control, but INT-767 outperformed OCA at both matched and potency-adjusted doses^18^. INT-767 also demonstrated higher potency, compared with OCA, in the modulation of FXR target genes in an *in vitro* system^30^. At variance with these observations, in our model the effects of INT-767 on collagen reduction was less pronounced and lasting compared to OCA, although INT-767 induced a dose and time-dependent reduction in collagen production.

The amount of extracellular collagen content is the result of the balance between its synthesis and turnover. Current evidence indicates that extracellular matrix (ECM) remodeling has a major pathological role in the progression of liver fibrosis, mediated by an alteration in metalloproteinase (MMP) activity within the liver^31^. Gelatinases (MMP-2 and MMP-9) are proteolytic enzymes able to degrade collagen as well as other extracellular components. They are secreted to the extracellular space in form of zymogens (proenzymes) and both their activation and inhibition are finely regulated. In hepatic chronic damage, as in NAFLD, gelatinase expression increases as well as their natural inhibitors TIMP1 and 2^31,32^. Thus, the regulation  of  MMP/TIMP  balance  is  essential  for  the  transition  of  physiological  ECM  into  a  pathological  one. When we assessed the activity of free/active gelatinase in the extracellular environment, 96 hours of FFA exposure induced a 30% reduction of gelatinase activity. Treatment with OCA was able to induce a clear dose-dependent increase of MMP-2 and 9 activity at 96 hours, while at 144 hours, despite a clear reduction in collagen content, the treated samples showed a trend towards a reduction in MMP-2 and 9 activity compared to FFA-treated samples. In chronic liver injury, dynamically regulated MMPs and TIMPs expression lead to a positive feedback loop with subsequent fibrogenesis^31^. Based on these results, we hypothesize that in our model 96 hours is the most appropriate time point to evaluate the effect of tested compounds on the modulation of gelatinases.

When activated, FXR forms a heterodimer with RXR to modulate expression of target genes. In addition to direct target genes, FXR modulates a number of genes through the regulation of SHP^33^. In this study, we evaluated the effect of FXR activation on FXR and SHP gene expression. We observed only a slight increase in SHP mRNA expression after prolonged exposure to FFAs, in line with a previous study reporting that saturated fatty acids, as palmitic acid, do not activate FXR^34^. Co-treatment with OCA induced a clear dose- and time-dependent decrease of FXR expression and an opposite trend in SHP with the exception of the highest concentration at the longest time point. Interestingly, treatment in the absence of FFAs induced a lower reduction of FXR that was comparable among samples, and a greater increase of SHP mRNA (between 5 and 15 fold of increase compared to FFAs treated sample), suggesting that the physiological state of the system can influence the effect of FXR agonists. Likewise, co-treatment with FFAs and INT-767 induced a progressive decrease of FXR expression and a slight increase of SHP, while in the non-NASH environment INT-767 induced a modest reduction of FXR mRNA and a greater increase in SHP expression compared to the FFA-treated samples (6.4 times higher compared to control). At variance with previous studies^16,30^, in the present study, OCA was more efficient in inducing FXR target genes compared to INT-767.

Globally, the data we obtained indicate that OCA exerts more potent antifibrotic effects compared to INT-767, conceivably due to its capacity to modulate gelatinase activity and promote collagen degradation.

The discrepancy between comparative analysis in this *in vitro* system and previously reported animal studies could be related to the fact that INT-767 is a dual FXR-TGR5 agonist. TGR5 is a classic G-protein coupled cell surface receptor, widely expressed in various tissues including gallbladder, bile ducts, adipose tissue, spleen, intestines, and kidneys. Within the liver, TGR5 is abundantly expressed in Kupffer cells and endothelial cells, but not in hepatocytes^35^. Thus, the absence of TGR5 in our system could explain the reduced efficacy of INT-767. Moreover, it was recently demonstrated, in murine models, that in the intestine FXR can induce TGR5 gene expression, which stimulates glucagon-like peptide-1 (GLP-1) secretion. Therefore, INT-767 could induce a general improvement in hepatic glucose and lipid metabolism in high-fat-diet-induced NASH mice, with a consequently increased efficacy in NASH improvement compared to OCA^16^. Interestingly, the authors observed that while INT-767 was more effective in improving glucose tolerance and hepatic insulin signaling, OCA was more effective in improving the hepatic lipid metabolism. This observation highlights the point that FXR agonist potency is not the only parameter to be considered when evaluating the efficacy of a new drug, as the downstream effects can differ among molecules.

Several experimental models have been characterized in an attempt to reproduce the molecular mechanisms involved in the onset of NAFLD and the progression of NASH to be used as preclinical models. *In vitro* cultures of hepatocytes and HSC have been historically based on preparations of primary cells isolated from rat liver. However, for the prediction of drug toxicity or to test drug-induced fibrosis in humans, the use of primary human hepatocytes or nonparenchymal cells is preferred since liver toxicity in humans shows poor correlation with animal studies^36^. The yield of hepatocytes isolated from human donors is scarce; the procedure is highly variable among preparations and depends on the condition of the donor’s liver. As an alternative, cell lines are readily available, easy to handle, phenotypically stable, low cost and have (almost) unlimited lifespan. The use of co-culture systems, such as hepatocytes and HSC, represents a valid platform for the study of cell cross-talk during fibrogenesis in the context of NAFLD. Indeed, several studies have shown a superior correlation to *in vivo* cellular phenotypes by the use of co-cultures rather than monoculture systems^37^. These 2D systems, have been proven to be valid for the assessment of cellular behavior but have limitations in maintaining the characteristics observed in the normal liver 3D microenvironment. The available 3D systems are not always easy to handle, and the experimental set-up has not been well standardized yet, thus limiting their use among different laboratories^38^. Conversely, the model used in this study is easy to handle, provides controlled conditions, and offers clear and straightforward information about compound efficacy on fibrogenesis in a NASH environment. These characteristics make our system an useful tool to screen for drugs acting on many mechanisms of NASH development. Thus, we tested in our system two other molecules currently being evaluated in ongoing clinical trials, a nonsteroidal FXR agonists, tropifexor, and an apoptosis signal-regulating kinase 1 (ASK-1) inhibitor (selonsertib), and compared their effects with OCA and INT-767.

Tropifexor is a nonsteroidal FXR agonists that derives from the modification of GW4064 to overcome its poor intestinal absorption^8^. So far, tropifexor has progressed into two phase II clinical trials. The FLIGHT-FXR study will assess its safety, tolerability, and efficacy on markers of liver inflammation in patients with NASH^39^, while the second one (TANDEM study) will evaluate its efficacy in combination with cenicriviroc in patients with NASH and liver fibrosis^40^.

Injured hepatocytes, undergoing apoptosis, are the hallmark of NASH and fibrosis progression^41^. In the context of oxidative stress, activation of ASK1, a serine/threonine signaling kinase, can induce the activation of stress response pathways worsening hepatic inflammation, apoptosis, and fibrosis^42^. Therefore, for the treatment of NASH the inhibition of ASK1 represents a good target. Selonsertib, a selective ASK1 inhibitor, was reported to significantly improve metabolic parameters and to reduce hepatic steatosis, inflammation and fibrosis in a murine model of NASH^43^. Following the results obtained in a phase 2 clinical trial evaluating the antifibrotic properties of selosertib in patients with NASH and moderate to severe fibrosis^44^, this inhibitor is now under evaluation in two phase 3 trials in adults with NASH and bridging (F3) fibrosis (STELLAR-3)^45^ or with compensated cirrhosis (STELLAR-4)^46^. However, recent results show that both STELLAR-4, and STELLAR-3 did not meet the pre-specified week 48 primary endpoint of a ≥1-stage histologic improvement in fibrosis without worsening of NASH^47,48^.

In our NASH model, both tropifexor and selonsertib reduced collagen deposition. Tropifexor was more effective at lower concentrations and after prolonged exposure, while all the tested selonsertib concentrations were able to decrease significantly and to a similar extent the extracellular collagen deposition at 96 hours, but this effect was lost over time. The gelatinase analysis showed no modulation of their activity after treatment, suggesting that these compounds act on extracellular matrix reorganization through different pathways or possibly with a different timing compared to semisynthetic bile acid derivatives.

In conclusion, in the present study we showed that the *in-vitro* model of NASH provides a promising tool for investigating efficacy of candidate drugs in NASH, in particular on liver fibrogenesis. As shown in Table 1, all the compounds tested induce a significant reduction of collagen deposition, with a different concentration range and timing. OCA was the most effective in reducing fibrosis, decreasing collagen by 38 to 54% compared to FFA-treated control, followed by INT-767 (30% reduction), selonsertib (14 to 36% reduction), and tropifexor (20% reduction).

**Table 1:** Modulation of collagen biosynthesis by anti-fibrotic drugs (FXR agonists or ASK1 inhibitor).

Compound	Concentration	Collagen reduction (%) *vs*. FFAs treated sample			
	[µM]	96 hours	*p-value*	144 hours	*p-value*
**OCA**	0.1	−1		+34	
	1	−**38**	*	−15	
	10	−**53**	**	−**54**	**
**INT-767**	0.1	−3		+20	
	1	−**32**	*	−4	
	10	−29		−29	
**Tropifexor**	0.1	−16		−**21**	*
	0.5	−14		−**14**	*
	1	−**36**	**	−1	
**Selonsertib**	0.05	−**33**	**	−16	
	0.1	−**35**	**	−1	
	0.5	−**33**	**	+11	

*p < 0.05; **p < 0.01 vs FFAs treated samples.

## Supplementary information

Supplementary Figure S1.
