# Supplementary material for: Obeticholic acid and INT-767 modulate collagen deposition in a NASH in vitro model
Source: Sci Rep. 2020 Feb 3;10:1699. doi: 10.1038/s41598-020-58562-x (PMC6997404; doi:10.1038/s41598-020-58562-x)
Supplement: Supplementary file 1 — Supplementary Figure S1. [file 41598_2020_58562_MOESM1_ESM.pdf]

TITLE: Obeticholic acid and INT-767 modulate collagen deposition in a NASH *in vitro* model

RUNNING TITLE: Effects of OCA and INT-767 in a NASH in vitro model

AUTHOR: Beatrice Anfuso[1], Claudio Tiribelli[1], Luciano Adorini[2], Natalia Rosso[1*]

AFFILIATIONS:

1. Fondazione Italiana Fegato, AREA Science Park Basovizza, SS14 km 163.5, 34149 Trieste, Italy.

2. Intercept Pharmaceutical, Inc. 10 Hudson Yards 37th Floor, 10001 New York, NY USA.

***Corresponding author**. Address: Fondazione Italiana Fegato, AREA Science Park Basovizza, SS14 km 163.5, 34149 Trieste, Italy. Tel.: +39 0403757922. Fax.: +39 0403757832.

E-mail address: natalia.rosso@fegato.it

A

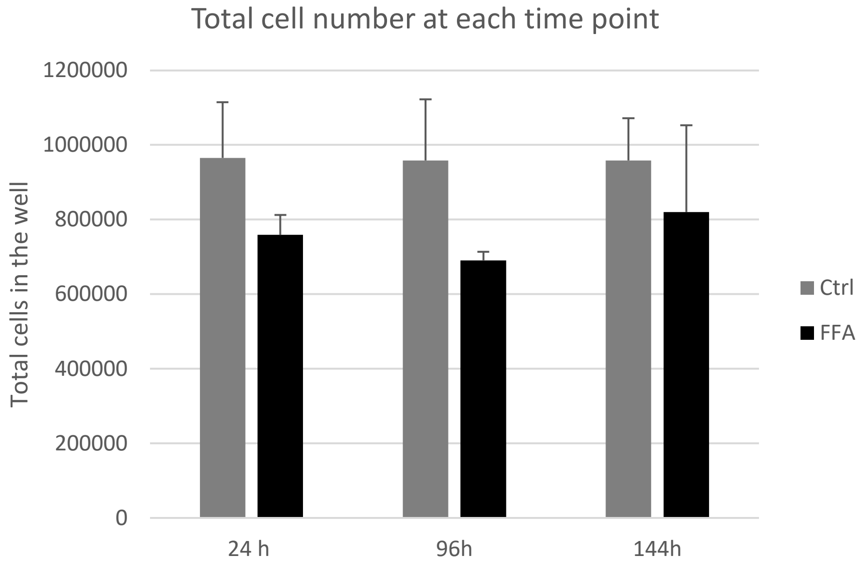

B

Total cell number in each well (n=3)

		mean	sd	p value
**24h**				
	CTRL	965,152	149,620	*0,99*
	FFA	758,824	53,414	*0,55*
**96h**				
	CTRL	958,666	164,065	*0,99*
	FFA	690,666	22,744	*0,55*
**144h**				
	CTRL	958,000	113,260	*0,99*
	FFA	820,000	232,559	*0,55*

C

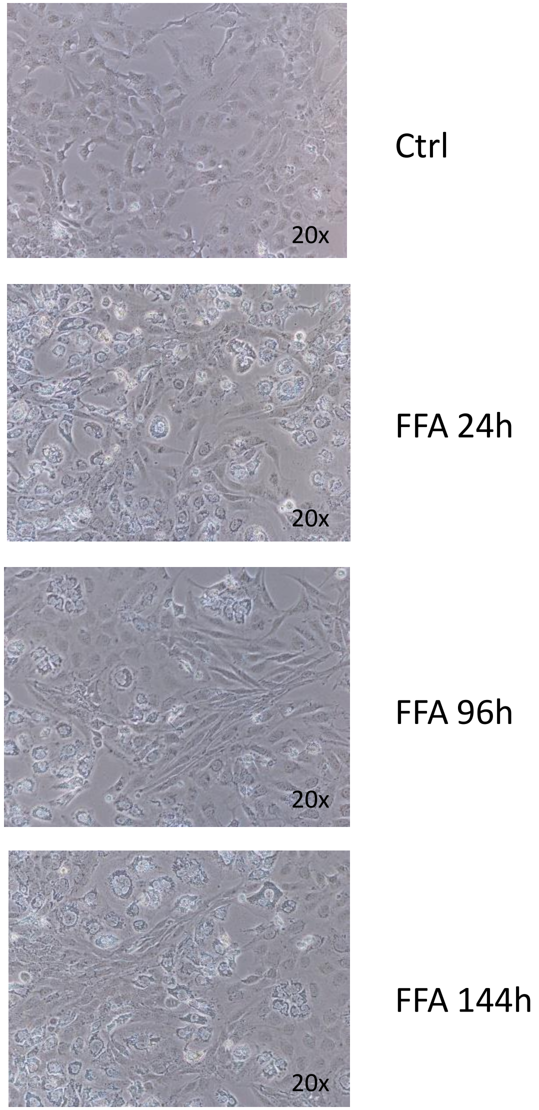

Supplementary Figure S1- A) Cell count in each well at the end of each experimental time point both for Controls (Ctrl) (grey) and FFA (black) **B)** Summary of the mean cell number ± SD of three independent biological replicates with the respective p value within each condition at all the time points **C)** Representative pictures of the cellular confluency at each experimental time point (20x bright field).
